# Autoimmune rheumatic diseases: One or many diseases?

**DOI:** 10.1016/j.jtauto.2021.100129

**Published:** 2021-10-28

**Authors:** Haralampos M. Moutsopoulos

**Affiliations:** National and Kapodistrian University of Athens and Academy of Athens, Vournazou 29, Athens, 11521, Greece

**Keywords:** Autoimmune rheumatic disease, Overlapping clinical syndrome, Common humoral auto-reactivity, Genetics, Therapeutics, Autoimmune rheumatic diseases, ARDs, Systemic lupus erythematosus, SLE, Rheumatoid arthritis, RA, Scleroderma, SScl, Sjögren's syndrome, SS, Mixed connective tissue disease, MCTD, Small nuclear ribonucleoprotein, sn RNP

## Abstract

Until the etiopathogenic factor(s) of autoimmune and autoinflammatory rheumatic disorders will be identified, their classification into entities will continue. However, their similar clinical manifestations, overlapping syndromes, evolution from one entity into another, as well as common autoantibody responses, suggest that autoimmune and autoinflammatory disorders may constitute distinct pathophysiologic processes on the basis of a different genetic background. Prognosis and effective therapeutic regimens are mostly based on the clinico-pathologic severity of the involved tissues or organs and not on the disease label.

## Highlights

• Autoimmune rheumatic diseases (ARDs) can evolve from one into another disease.
• Different ARDs can appear in the same individual.
• Common humoral auto-reactivities appear in different ARDs.
• Therapy is based on the severity of ARDs expression.

## 1 Introduction

Autoimmune rheumatic diseases (ARDs), is a group of distinct disorders, which share similar clinical, laboratory and immunological manifestations. Their fundamental patho-biological finding is the unfolding of an excessive self-reactive, antigen-driven, immune response. On the basis of clinical and translational research studies, it is widely accepted that their pathogenesis is the result of genetic predisposition [1], environmental insults such as infections, chemical and physical agents, hormonal alterations [2], as well as stressful life events [3,4].

Based on the presence or absence of a proinflammatory response, ARDs are classified into three groups: autoinflammatory (e.g., ankylosing spondyloarthritis), autoimmune [e.g., systemic lupus erythematosus (SLE)], and those with overlapping features, with rheumatoid arthritis being the best example (Table 1a, Table 1ba and 1b). Irrespectively of the specific disease type, the affected organs are infiltrated by auto-reactive and activated immunocytes and their chemical products proinflammatory cytokines [5]. In autoimmune disorders, in addition to auto-activated lymphocytes, autoantibodies alone or in conjunction with circulating or tissue autoantigens, along with activation of type I interferon pathways are prevalent [[6], [7], [8]].

**Table 1a:** The main autoimmune and autoinflammatory rheumatic diseases.

**Autoimmune**	**Autoinflammatory**
Systemic Lupus Erythematosus	Rheumatic fever
Sjögren's syndrome	Post-infectious arthritis (Reiter's syndrome)
Systemic scleroderma	Psoriatic/Enteropathic Arthritis
Mixed connective tissue disease	Ankylosing Spondylitis
ANCA + Vasculitis	Adult-onset juvenile arthritis
Relapsing polychondritis	Periodic Fever Syndromes
Inflammatory muscle diseases	Large vessel vasculitis
	Polyarteritis nodosa
**Overlapping autoimmune/autoinflammatory**	
Rheumatoid arthritis	
IgG4 related diseases

**Table 1b:** Major differences among autoimmune and autoinflammatory diseases.

**Autoimmune**	**Autoinflammatory**
Autoantibodies and tissue infiltration by activated immune cells	Tissue infiltration by activated immune cells
Absence of acute-phase proteins	Elevated acute-phase proteins
Activation of type I Interferon pathways	Increased pro-inflammatory cytokines (IL1, 6, 17, TNF)
**Overlapping autoimmune/autoinflammatory diseases**	
Autoantibodies and tissue infiltration by activated immune cells	
Increased pro-inflammatory cytokines (IL1, 6, 17, TNF)	

Are these entities different or each one of them represents a piece of the “puzzle” called today autoimmune/autoinflammatory diseases? Till the etiologic factor(s) responsible for their generation are identified, classification into individual entities will continue.

Taken together, the shared clinical manifestations between apparently distinct nosological entities, overlapping disease features in the same individual, evolution from one disorder into another during follow-up, as well as the implementation of non-disease specific therapies, promoted the formulation of a hypothesis suggesting that these disorders may be different clinical expressions of the same or similar etiopathologic factor(s).

## 2 Clinical paradigms

### 2.1 Clinical manifestations

The patho-biologic process of ARDs affects primarily a target cell and/or human tissue. Rheumatoid arthritis (RA) has a predilection for the articular synovia; SLE, scleroderma (SScl) and vasculitis affect the vascular endothelium of different organs, while the activated immunocytes insult the secretory epithelia of Sjögren's syndrome (SS) exocrine glands, renal tubules, cholangia and bronchi. On the basis of these clinical-pathological manifestations, the term autoimmune epithelitis was proposed for SS [9]. Studies of cultured isolated salivary gland epithelia from SS patients have shown that they are activated, can act as antigen-presenting cells and release autoantigens through apoptotic blebs and exosomes [10].

Beyond the specific manifestations of a given entity, ARDs share several common clinical manifestations. In more detail, arthralgia/arthritis, serositis, Raynaud's phenomenon, interstitial lung disease, and glomerulonephritis are among the commonest, occurring with different frequencies among ARD entities. In Table 2 the incidence of Raynaud's phenomenon is shown in different ARDs. This nonspecific disease manifestation, as well as others, e.g., arthritis, can precede the full-blown development of a specific ARD by many years. On clinical grounds, classification of an ARD can be sometimes difficult or not possible. For instance, a patient presenting with keratoconjunctivitis sicca, xerostomia, membranoproliferative glomerulonephritis, leukopenia, antinuclear antibodies, anti-Ro/anti-La autoantibodies and cryoglobulins, will be called by some specialists as systemic (extra-glandular) Sjögren's syndrome, while for others the diagnosis of Sjögren's syndrome associated with systemic lupus erythematosus is undertaken. Is it important how this syndrome is called in the everyday practice? Apparently NOT, since both specialists will treat this patient with systemic disease in the same manner: Intravenous monthly pulses of glucocorticoids and cyclophosphamide or combination of steroids with mycophenolate mofetil. Another case difficult to be classified is in an individual, more often a woman, who presents with arthralgia of the small and large joints and, upon laboratory testing, antinuclear autoantibodies (ANA) and anti-Ro-52 autoantibodies are detected in the serum. She will be called as suffering from undifferentiated connective tissue disease and will be treated with hydroxychloroquine as arthralgia is treated in patients with SLE or SS.

**Table 2:** Incidence of Raynaud's phenomenon in different ARDs.

Disease	Incidence (%)
Scleroderma	85–90
Mixed connective tissue disease	90–100
Systemic lupus erythematosus	30–40
Sjögren's syndrome	30–40
Rheumatoid arthritis	<10

Since ANA and antiRo-52 autoantibodies can be found in many ARDs (see below), long follow-up may reveal the disease entity the patient suffers from Ref. [11].

### 2.2 Prognosis of ARDs

Prognosis of ARDs is not based on the disease label but on the presence of certain clinical and or laboratory findings. For example, in RA patients, high disease activity, early development of joint erosions, high titers of rheumatoid factors and anti-citrullinated autoantibodies, and extra-articular manifestations are poor prognostic factors [12]. SLE patients with a younger age of onset and proliferative glomerulonephritis have a worse prognosis [13]. In patients with Sjögren's syndrome, parotid gland enlargement, purpura, leukopenia, low C4 levels, presence of circulating rheumatoid factors, cryoglobulins, autoantibodies to Ro/La autoantigens and Interferon -stimulated gene 15 in the peripheral blood and in the labial minor salivary glands, constitute risk factors for B-cell lymphoma development [14,15]. In patients with microscopic polyangiitis, pulmonary fibrosis may precede other disease manifestations by a variable length of time, while they may have a poor prognosis and increased mortality [16].

### 2.3 Overlapping ARD features in patients

Overlapping clinical features have been seen in some ARD patients. The characteristic example of an overlap syndrome is the mixed connective tissue disease (MCTD). Patients with this disorder manifest with clinical features “typical” of different ARDs (SLE, myositis, RA, SScl and SS), without fulfilling classification criteria of any of the aforementioned ARDs. MCTD patients have in their sera high titers of autoantibodies to small nuclear ribonucleoprotein (sn RNP, or RNP) [17].

Another overlapping syndrome associated with a group of autoantibodies is the one presenting with features of rheumatoid arthritis, scleroderma, and myositis. In the sera of these patients, antibodies to t-RNA synthetases are detected. According to different studies these patients present often with interstitial lung disease and arthritis/arthralgia/myalgia. Over 40% of them suffer from Raynaud's phenomenon, while less often they develop fever and mechanic hands [18].

A third overlap ARD syndrome is characterized by the presence of autoantibodies against PM-Scl. These patients present with features of myositis, scleroderma, interstitial pulmonary disease and tendonitis [19].

### 2.4 Evolution to another ARD

Previous studies have shown that one fourth of RA patients, after a long follow-up, will develop features of SS [20,21]. On the contrary, some SS patients during their follow-up develop clinical and/or laboratory features of SLE [22]. In addition, during follow-up, some patients with seropositive RA, can develop SScl (sclerodactily, pulmonary hypertension, esophageal dysfunction, and cardiac involvement), SLE (photosensitivity reaction, nephritis, leukopenia, lymphopenia, thrombocytopenia, Coombs positive hemolytic anemia, and positive anti-dsDNA), as well as features reminiscent of SS [23].

We have recently described a patient suffering from SScl, who suddenly presented clinical and laboratory manifestations of eosinophilic fasciitis, (cutaneous “peau d'orange” appearance, peripheral blood eosinophilia) and inflammatory myositis (muscle pain and weakness, elevated muscle enzymes). All three entities were documented by a through and through biopsy from skin-fascia to muscle [24].

## 3 Lessons from genetic and epigenetic studies

If ARDs represent distinct clinical expressions of similar or the same etiopathologic agent(s), how can this diversity is explained? Genetic factors play a significant role in their pathogenesis. Indeed, the high prevalence of autoimmune and autoinflammatory disorders among family members are highly suggestive of their genetic predisposition. On the other hand, the high concordance in identical twins (typically between 25% and 50%), being 10 times higher compared to fraternal twins (typically between 2% and 8%) [25], implies the significant role of environmental contributors in ARDs pathogenesis, as well. Moreover, the role of genetic factors is attested by the HLA alloantigen association's studies with different ARD susceptibility. More specifically, ankylosing spondyloarthritis, and enteropathic and psoriatic arthritis are all associated with the alloantigen HLA-B27 in different frequencies. SLE patients are associated with HLA-DR2 and HLA-DR15; SScl also with HLA-DR15; SS with HLA-DR3 and HLADQA1*0501, and RA with HLA-DR4 and HLADRB1DQA1-DQB. Furthermore, genome association studies have revealed that autoimmune disorders are associated with the same or different genes involved in various immune pathways (Table 3) [26]. These observations suggest that clinical phenotypes of different ARDs are strongly influenced by the genetic background in a counterplay with environmental triggers.

**Table 3:** Paradigms of genes associated with ARDs.

Rheumatoid arthritis	Common			Different
	IRF5	CTLA4	STAT4	IL-23 R
Systemic lupus erythematosus	>	>	>	PBCD1
Scleroderma	>	>	>	TNFS4
Sjogren's syndrome	>	>	>	BAFF

*ARDs*:*Autoimmune Rheumatic Diseases*.

*CTLA4:Cytotoxic T Lymphocyte Associated Protein 4*.

*STAT4:Signal Transducer and Activator of transcription 4*.

*IL-23R:Receptor of Interleukin 23*.

*PBCD1:Programmed Cell Death 1*.

*TNFSF4:Tumor Necrosis Factor Superfamily member 4*.

*BAFF:B Cell Activating Factor*. IRF5: Interferon Regulatory Factor 5.

More recent data in salivary gland and kidney biopsies from SS and SLE patients respectively have shown that epigenetic alterations, such as defective methylation of endogenous retroviral-elements at tissue level, as a result of altered expression of methylating enzymes, could lead to overexpression of endogenous retroviral-elements and activation of type I interferon pathways in these patients; these observations could imply that tissue-specific epigenetic alterations could account for the diversity observed among ARDs [27,28].

## 4 Etiologic agents as initiators of ARDs

Different studies have shown that certain viruses can trigger the development of different autoimmune-like disorders. More specifically, HIV virus can produce in some infected individuals SS-like disease and cryoglobulinimic vasculitis [29,30]. A similar autoimmune picture can be developed in patients with chronic hepatitis C virus infection [31]. Recently, the SARs–CoV2 virus was shown to induce in some individuals autoimmune disorders, such as antiphospholipid syndrome, and Kawasaki or Guillen Barre syndromes [32]. The best examples of ARDs induced by microorganisms are rheumatic fever and post-infection arthritis, previously known as Reiter's syndrome. Despite these observations, a definite viral etiological factor has yet to be identified in the majority of ARDs. However, herpes, Epstein-Bar and Coxsakie viruses have been implicated as major triggers of some ARDs. These viruses can initiate autoimmunity through several mechanisms such as molecular mimicry, epitope spreading, bystander activation and/or immortalization of infected B cells [[33], [34], [35], [36]]. Recently, autoimmune encephalitis developed in a patient infected with the West Nile Virus. In his serum anti-glycine receptor antibodies, were detected. An *in silico* analysis revealed certain sequence similarities between Nile viral antigens and glycine receptor sequence fragments, suggesting that molecular mimicry between the viral antigens and autoantigens were the major players in autoimmunization [37].

In contrast, a growing body of evidence supports the protective role of viruses against the development of autoimmunity [38]. These opposite effects of viral infections on autoimmunity are influenced by different host genetic-virus interactions. Further molecular virology research is necessary to delineate the viral-host- interactions in ARDs and to provide a clear patho-biologic basis of how a viral infection can trigger the development of an autoimmune disease in a genetically predisposed individual.

## 5 Lessons from the autoantibody profile in ARDs

Autoimmune diseases are characterized by B-lymphocyte hyper-reactivity, as attested by hyperglobulinemia and a plethora of autoantibodies directed against circulating, membrane-bound and cellular and organ specific autoantigens. Some autoantibodies are disease-specific (anti-dsDNA and anti Sm for SLE; anti-La for SS, anti-Scl70 for SScl, and, anti-tRNA synthetases for a specific group of myositis), while anti immunoglobulins (Rheumatoid factors), anti- snRNP, anti-Ro52 and anti-Ro60 autoantibodies can be detected in the sera of patients with different ARDs (Table 4) [39].

**Table 4:** Distribution of ARDs in autoantibodies to Ro52, Ro60 and both Ro52 and Ro60 positive sera.

Disease	Anti-Ro52 (%)	Anti-Ro60 (%)	Anti-Ro52 and anti-Ro60 (%)
SS	21	13	66
SLE	14	28	58
UCTD	38	15	46
RA	56	11	33
IMM	82	–	18
PBC	78	11	11
SCLE	25	37	37

UCTD: Undifferentiated connective tissue disease; IIM: Idiopathic inflammatory muscle diseases; SCLE: Subacute cutaneous lupus.

The study of autoantibody profiles during the patient evaluation, however, can be a useful diagnostic and prognostic tool for the ARD classification, allowing the definition of disease subtypes with different clinical manifestations [40]. Furthermore, autoantibody profile may precede by many years the development of a given autoimmune disease [41]. So far an effective therapeutic intervention to halt the clinical development of an ARD in individuals with solo autoantibody reactivity is not available. Similar humoral reactivity against some autoantigens in different ARDs, further supports the notion that these disorders may share similar etiopathogenic mechanisms.

## 6 Lessons from the therapeutic interventions

Thanks to the progress in our understanding of the patho-biologic mechanisms responsible for the clinical expression of ARDs, the medical community currently possesses an array of effective therapeutic agents, including antimetabolites, JAK inhibitors, cytophiline binders, anti-pro inflammatory biologic therapies, such as anti-TNFα, anti-IL-1, 6, 17, and anti-B cell therapies. These therapeutic agents suppress auto-reactivity, prevent and sometimes reverse the tissue injury produced either by the activated immunocytes and their products (metalloproteinases, cytokines and chemokines) or by autoantigen-autoantibody complexes, which activate the complement cascade, further fueling the inflammatory process.

The application of a given therapeutic agent, however, is not dictated by the label of a given entity, but by the predominant clinical manifestation(s) and their severity. For example, arthritis, regardless of the underlying disease, is treated with methotrexate alone or in combination with anti-proinflammatory biologic agents [42], glomerulonephritis is treated with similarly effective medications, the mycophenolate mofetil or the intravenous monthly pulses of cyclophosphamide, both in combination with high methyl prednisolone doses [43].

## Declaration of competing interest

The authors declare that they have no known competing financial interests or personal relationships that could have appeared to influence the work reported in this paper not have resieved any funds for this work.
